# Computer Navigation-Assisted Resection of Heterotopic Ossification Around the Hip: A Technical Note

**DOI:** 10.7759/cureus.42897

**Published:** 2023-08-03

**Authors:** Mohamed Amine Selmene, Peter Upex, Mourad Zaraa, Pierre Emmanuel Moreau, Guillaume Riouallon

**Affiliations:** 1 Orthopaedic Surgery, Paris Saint-Joseph Hospital Group, Paris, FRA

**Keywords:** pelvis, femur, hip, ct-guided surgery, surgical navigation systems, heterotopic ossification

## Abstract

Heterotopic ossification is a rare but debilitating situation. It occurs in patients who have undergone paralysis and/or immobilization. Hip osteoma is one of the most frequent locations and is associated with a significant functional handicap. Its treatment is based on surgical resection, which is a risky surgery that is not devoid of complications such as infections, hematoma, and recurrence. We describe in this paper a new surgical technique that adds to the classic hip osteoma resection: guidance with a navigation system coupled to a 3D imaging tool. We performed this technique on two patients (three hips, one bilateral case). We think that this technique makes the surgery safer with fewer complications.

## Introduction

Heterotopic ossification (HO) is an ectopic ossification in soft tissues around a joint. This bone development occurs in patients who sustained trauma or a major neurologic injury. The hip is one of the most affected joints [1]. It can cause significant pain and a reduced range of motion, which leads to a marked impairment in quality of life.

Therefore, surgical resection of HO is necessary and sometimes urgent, especially for restoring joint mobility, sitting, and walking, as well as for nursing. This type of surgery is not without risks such as bleeding, prolonged operating time, infections, and recurrence [2,3], and should be planned and performed appropriately.

So far, HO resection has been based solely on preoperative planning using CT scans. We present a new surgical technique for hip osteoma resection using the CT-guided navigation system with intraoperative planning and guidance, which can make surgery simpler and more prudent.

## Technical report

Installation

The patient can be installed prone, supine, or lateral. This will depend on the location of the osteoma and the approach. A traction table or a radiolucent (carbon fiber) plateau can be used. This makes it easier to set up the imaging apparatus. The intraoperative guidance is performed using a navigation system coupled with 3D imaging (here, the O-arm® Intra-operative Imaging System with StealthStation® Navigation, Medtronic was used).

**Figure 1:**
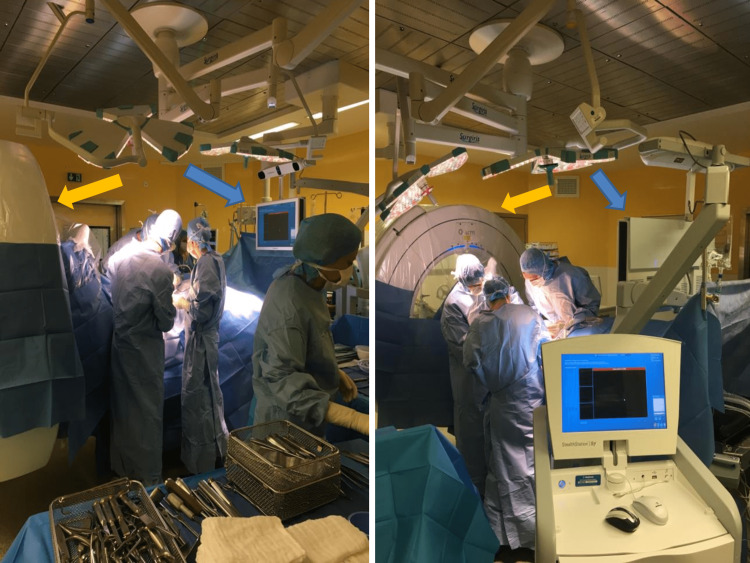
Example of a patient installation. The O-arm® is placed in the parking position on the side of the patient’s feet (yellow arrow). The navigation screen is placed on the other side, near the patient’s head (blue arrow).

The imaging apparatus is positioned according to anteroposterior and lateral centering, then ‘parked’ at the patients’ feet. The navigation screen is installed near the patient’s head (Figure 1).

Sterile draping includes the area where the surgical approach was decided (anterior, lateral, posterolateral, Kocher-Langenbeck, Stoppa approach, etc.). This could include either the upper third of the anterior region of the thigh to the umbilicus or the posterior region from the upper third of the thigh to the iliac crests.

Procedure

The surgery begins with a 3D image acquisition after positioning the reference frame with passive localizers fixed with two Kirschner wires (K-wires), either on the anterosuperior iliac spine (when the patient is in supine or lateral position) or on the posterosuperior iliac spine (when the patient is in prone position).

After the surgical approach, a navigation pointer shows the resection boundaries, which are the iliac and femoral implantations of the HO. The HO is exposed progressively by releasing the covering muscles with an electric cautery to reduce bleeding. We perform a bone chisel cut on the HO implantation bases, and we continue the exposure of the osteoma in order to excise it en bloc in the supracapsular cleavage plane. This resection is carried out with meticulous control of nervous and vascular structures. Careful hemostasis is performed, and two Redon drains are let into the dead zones of resection.

A two-dimensional or three-dimensional acquisition is performed at the end of the procedure in order to ensure the absence of residual osteoma that should be removed.

Patients

**Figure 2:**
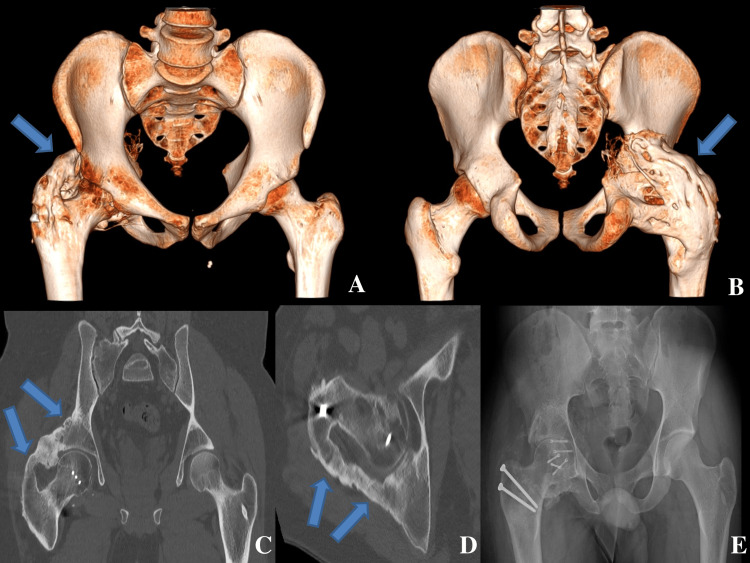
Pre- and postoperative imaging of the first patient A and B: preoperative pelvis 3D-scan image; C: coronal view of the preoperative pelvis multiplanar reconstruction (MPR); D: axial view of the preoperative pelvis MPR; E: postoperative pelvis X-ray

We performed this technique on two patients (three hips). The first case was a 24-year-old man who was the victim of polytrauma following a motor vehicle accident. He had a right acetabulum and femoral head fractures. An open reduction and internal fixation of these fractures by screws were performed. He developed a posterior osteoma of this hip one year later. He was operated on via the posterolateral hip approach (Figure 2).

**Figure 3:**
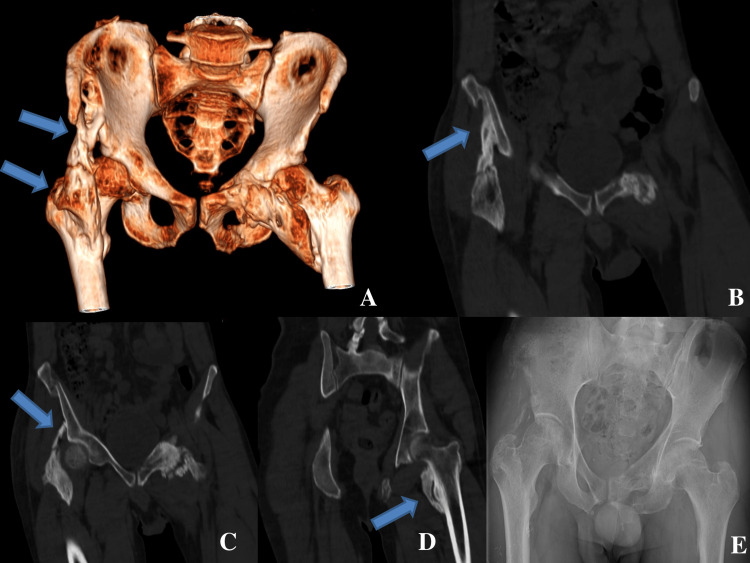
Pre- and postoperative imaging of the second patient A: preoperative pelvis 3D-scan image; B: right hip osteoma: proximal iliac implantation; C: right hip osteoma: distal iliac implantation, left hip osteoma: proximal implantation on the iliopubic branch; D: left hip osteoma: femoral implantation; E: pelvis X-ray at the final follow-up

**Figure 4:**
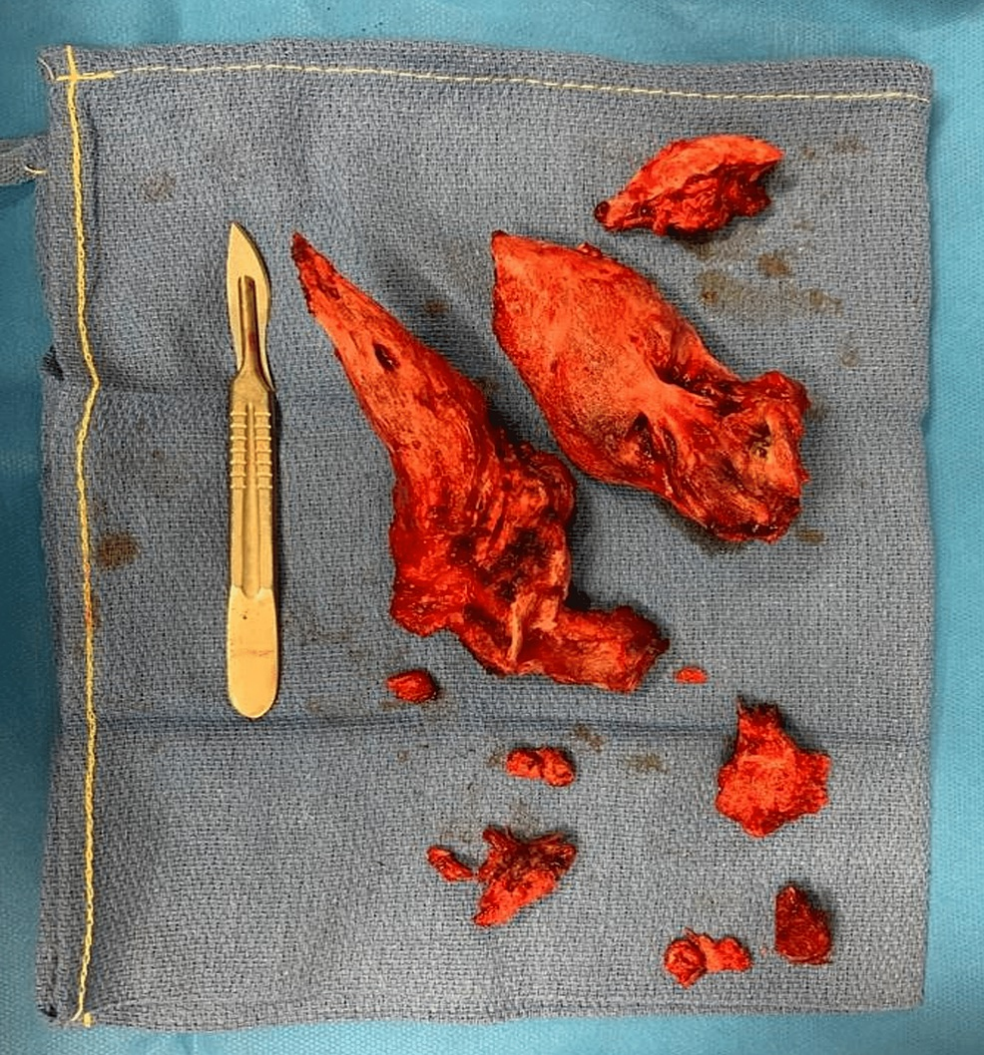
En bloc resection of the two osteomas of the second patient

The second case was a 40-year-old man who was a polytraumatized patient following a defenestration. He had pelvis, sternum, scapula, right humerus, elbow, and left calcaneus fractures. He had a hepatic artery embolization and was operated on for ischemic cholecystitis. For all that, he was bedridden for more than two months and developed a bilateral hip HO. This patient was operated on on both hips at the same time. We performed the Smith-Peterson approach (iliofemoral or enlarged anterior hip approach) on the right side and the Hueter approach (anterior hip approach) to excise the femoral implantation, combined with a modified Stoppa approach (an extraperitoneal endopelvic approach) for the iliopubic branch implantation on the left side (Figures 3-4).

No perioperative incidents were noted. The two patients did not receive any blood transfusions during the immediate postoperative stay. No infections or recurrences were reported at a mean follow-up of 20 months.

## Discussion

Heterotopic ossification is an ectopic ossification in soft tissues around joints [4]. There are two forms of HO: a genetic and an acquired form [2,4]. It mainly affects the periarticular tissues of the hip [2,5-7]. The acquired forms of osteoma concern polytraumatized patients, who usually require a period of critical care for trauma or a major neurologic injury such as traumatic brain injury, spinal cord injury, stroke, and cerebral anoxia [5,6,8]. When it comes to the hip, HO also follows acetabular fractures or hip surgery (total hip arthroplasty) [9,10].

When HO becomes symptomatic, it is associated with pain, limited range of motion, a limitation of activities of daily living, especially in paraplegic patients, the inability to sit in a wheelchair, and limited personal hygiene, which is a high-risk factor for the appearance of bedsores [4,7].

The management of hip HO consists of surgical resection, which provides excellent postoperative results despite a high complication rate such as draining wounds, infections, hematoma, and recurrence [2,3,11,12]. A good preoperative CT scan plan is recommended to reduce the complication rate [12].

All these arguments push us to better plan this surgery in order to achieve an appropriate execution. Thereby, we present our new technique that, we think, could make this type of surgical management safer intra- and postoperatively.

To our knowledge, this is the first publication concerning HO resection using a CT-guided navigation system.

It is a technique that facilitates intraoperative management. It was reported in the literature that preoperative 3D-CT imaging, complemented in some cases by angiography, allows the surgeon to define the 3D anatomy of the HO accurately, their possible contact with the vascular-nervous elements, and plan the surgical excision with precision [13,14]. Therefore, we believe this technique, with its intraoperative 3D image acquisition, allows for precise and rapid surgery.

Concerning the surgical technique, Denormandie et al. reported that the iliac and femoral implantation bases have to be determined as the first step of the surgery [5]. They added that, for fear of increased morbidity, resection shouldn’t be exhaustive, unlike in oncologic surgery. Only the part of the heterotopic ossification that is causing problems should be removed. For example, during excision, the normal bony contour guides the resection and should be well exposed, especially in the femoral implantation, in order to avoid iatrogenic fractures [5,15]. In our technique, the navigation pointer makes it easier to go directly to the target, which is the implantation base of the HO. This allows for a quick and efficient bone chisel cut where it should be. It also allows an en bloc resection of the osteoma. On the other hand, heterotopic ossifications are always extra-articular and develop around joints. The capsule is always conserved and constitutes a cleavage plane [5,6]. This cleavage plane, whereby resection must be controlled, can be guided by navigation in our technique.

The use of navigation for this type of surgery allows us to perform exactly what was planned, thus shortening operating time. Consequently, this can decrease the rate of infections and intraoperative bleeding. Postoperative care will be more comfortable for patients, and rehabilitation will start as soon as possible. Moreover, it allows us to operate on both hips at the same time, just like we did with our second patient, considering that bilateral hip HOs are frequent [7].

However, like any surgical technique, it has limitations. This technique requires a specific technical platform. Surgeons must have a navigation system available in the operating room coupled with a 3D imaging tool and have a good command of it.

Finally, Stoira et al. recently published a paper stating a high prevalence of heterotopic ossification in critically ill patients with severe COVID-19 [16]. They reported that prolonged immobilization as a result of longer sedation and neuromuscular blockade for severe acute respiratory distress syndrome has played a decisive role in HO in their patients. However, it is plausible that other factors, such as systemic inflammatory conditions and local myositis, possibly due to the severe acute respiratory syndrome coronavirus 2 (SARS-CoV-2) virus, might have contributed to the higher prevalence of HO [2,17]. This technique may prove to be more necessary nowadays, apart from its other indications, because of the COVID-19 pandemic.

## Conclusions

Heterotopic hip ossifications occur in medically and psychologically fragile patients. They represent a turning point in the development of their illnesses. Their surgical management must be meticulous and effective.

Computed tomography-guided navigation, when available and well-commanded, may be of great help in the case of HO resection. This concerns intraoperative management and postoperative care. It’s a judicious technique and its results should be studied over larger series. This is part of our goals for the future.
